# *In vitro* inhibitory effects of kaempferitrin on human liver cytochrome P450 enzymes

**DOI:** 10.1080/13880209.2019.1656257

**Published:** 2019-08-28

**Authors:** Ning Zhang, Jing Liu, Zhixia Chen, Wenwen Dou

**Affiliations:** a Department of Neonatology, Yidu Central Hospital of Weifang, Weifang, Shandong, China;; b Department of Pediatric Medicine, Yidu Central Hospital of Weifang, Weifang, Shandong, China;; c Department of Orthopaedics, Yidu Central Hospital of Weifang, Weifang, Shandong, China;; d Department of Infectious Diseases, Affiliated Hospital of Weifang Medical University, Weifang, Shandong Province, China

**Keywords:** CYP1A2, CYP3A4, CYP2C9, Herb-drug interaction

## Abstract

**Context:** Kaempferitrinis (KF) is a bioactive flavonoid and possesses numerous pharmacological activities. However, whether KF affects the activity of human liver cytochrome P450 (CYP) enzymes remains unclear.

**Objective:** This study investigates the effects of KF on eight major CYP isoforms in human liver microsomes (HLMs).

**Materials and methods:** *In vitro*, HLMs were used to investigate the inhibitory effects of KF (100 μM) on the eight human liver CYP isoforms (i.e., 1A2, 3A4, 2A6, 2E1, 2D6, 2C9, 2C19, and 2C8), and corresponding probe substrates were used. Enzyme kinetic studies (0–50 μM of KF) were conducted to determine the inhibition mode of KF on CYP enzymes.

**Results:** The results showed that KF inhibited the activity of CYP1A2, 3A4, and 2C9, with IC_50_ values of 20.56, 13.87, and 14.62 μM, respectively, but that other CYP isoforms were not affected. Enzyme kinetic studies showed that KF was not only a noncompetitive inhibitor of CYP3A4, but also a competitive inhibitor of CYP1A2 and 2C9, with *Ki* values of 7.11, 10.24, and 7.58 μM, respectively. In addition, KF is a time-dependent inhibitor for CYP3A4 with *K_I_*/*K_inact_* value of 10.85/0.036 min/μM.

**Discussion:** The *in vitro* studies of KF with CYP isoforms indicate that KF has the potential to cause pharmacokinetic drug interactions with other co-administered drugs metabolized by CYP1A2, 3A4, and 2C9.

**Conclusion:** It is recommended that KF should not be used with other drugs metabolized by CYP1A2, 3A4, and 2C9. Further clinical studies are needed to evaluate the significance of this interaction.

## Introduction

**Figure 1.**
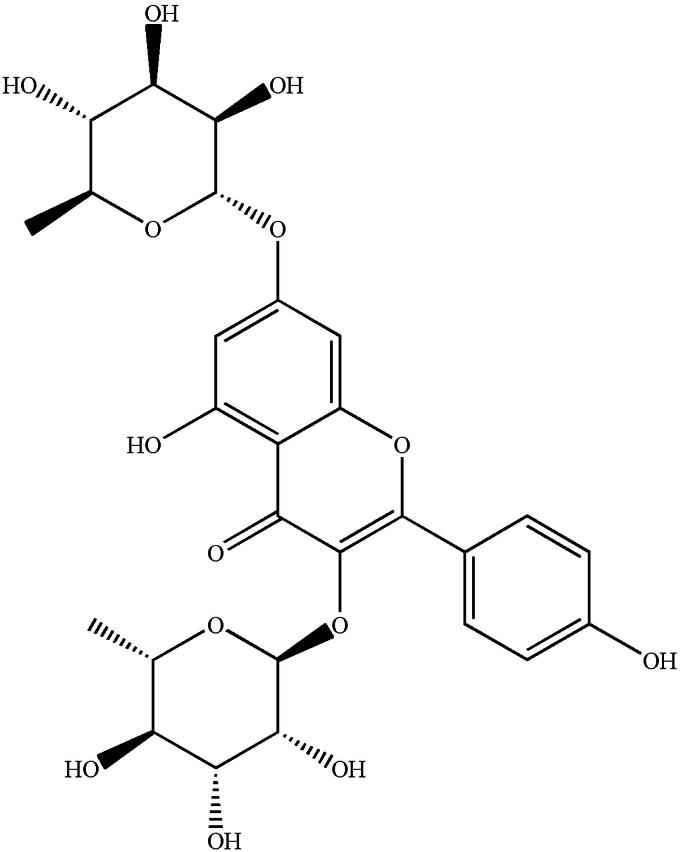
The chemical structure of kaempferitrin.

Kaempferitrinis (KF) (Figure 1) is a bioactive flavonoid isolated from aqueous extract of *Justicia spicigera* Schltdl (Acanthaceae), the leaves of *Bauhinia forficata* L. (Fabaceae), and the leaves of *Vepris heterophylla* (Engl.) Letouzey (Hamzah et al. 1994; Jorge et al. 2004; Gonzalez-Trujano et al. 2017). It is reported that KF can inhibit proliferation, induce apoptosis, and ameliorate inflammation in human rheumatoid arthritis fibroblast-like synoviocytes (Wang and Zhao 2019). Furthermore, KF exerts cytotoxic and antitumor effects against HeLa cells (Alonso-Castro et al. 2013) and shows anticonvulsant effects as potential natural products (Gonzalez-Trujano et al. 2017). In addition, KF shows a hypoglycemic effect as a consequence of the altered intrinsic activity of the glucose transporter (Jorge et al. 2004). In line with this, a comparative proteomic study of secretomes in kaempferitrin-treated CTX TNA2 astrocytic cells found that KF did not increase pro-inflammatory cytokine levels, which may have neural degenerative effect or aid in the progress of diabetes (Ku et al. 2017). Daniel Da Silva et al. (2014) found KF stimulated the glycolytic enzyme 6-phosphofructo-1-kinase (PFK) in a model of diabetes, and these findings suggest that KF may be a viable agent for the prevention and treatment of diabetic nephropathy (DN) (Jiang et al. 2018).

Cytochrome P450 (CYP) enzyme, a superfamily of heme-containing isoenzymes located primarily in hepatocytes, are important phase I enzymes in the biotransformation of xenobiotics, which include drugs, environmental pollutants, carcinogens and endogenous substrates (Wrighton and Stevens 1992; Yan and Caldwell 2001; Zhang et al. 2012). CYP1A, CYP2C, CYP2D, CYP3A and CYP2E are major CYP enzymes in drug metabolism (Li 2001; Zhang et al. 2017; Dong et al. 2018). *In vitro* P450 enzyme inhibition assays have been routinely used to assess the P450-mediated drug-drug interactions (DDI) potential of these enzymes. Most CYP enzymes can be inhibited or induced by a variety of drugs and chemicals that can give rise to toxicity or treatment failure. Therefore, the effects of KF on the activity of CYP enzymes should be investigated. To the best of our knowledge, few studies have investigated the effects of KF on CYP enzymes, particularly the inhibitory effects, which will increase the risk of therapeutic applications of KF and its medical preparations.

The purpose of this study was to investigate the effects of KF on eight major CYP isoforms in human liver microsomes (HLMs). *In vitro*, phenacetin (CYP1A2), testosterone (CYP3A4), coumarin (CYP2A6), chlorzoxazone (CYP2E1), dextromethorphan (CYP2D6), diclofenac (CYP2C9), *S*-mephenytoin (CYP2C19) and paclitaxel (CYP2C8) were used as probe substrates to determine the effects of KF on eight CYP enzymes. In addition, enzyme kinetic studies were conducted to determine the inhibition mode of KF on CYP enzymes.

## Materials and methods

### Chemicals

Kaempferitrin (≥98%) and testosterone (≥98%) were obtained from the National Institute for the Control of Pharmaceutical and Biological Products (Beijing, China). d-Glucose-6-phosphate, glucose-6-phosphate dehydrogenase, NADP^+^, phenacetin (≥98%), acetaminophen (≥98%), 4-hydroxymephenytoin (≥98%), 7-hydroxycoumarin (≥98%), 4′-hydroxydiclofenac (≥98%), sulfaphenazole (≥98%), quinidine (≥98%), tranylcypromine (≥98%), chlorzoxazone (≥98%), 6-hydroxychlorzoxazone (≥98%), paclitaxel (≥98%), 6β-hydroxytestosterone (≥98%), clomethiazole (≥98%), and furafylline (≥98%) were obtained from Sigma-Aldrich Co. (St. Louis, MO, USA). Montelukast (≥98%) was obtained from Beijing Aleznova Pharmaceutical (Beijing, China). Coumarin (≥98%), diclofenac (≥98%), dextromethorphan (≥98%), and ketoconazole (≥98%) were purchased from ICN Biomedicals (Aurora, Ohio, USA). Pooled HLMs (452161) were purchased from BD Biosciences Discovery Labware (North Carolina, USA). All other reagents and solvents were of analytical reagent grade.

### Assay with human liver microsomes

As shown in Table 1, to investigate the inhibitory effects of KF on different CYP isoforms in HLM, the following probe reactions were used, according to previously described method (Zhang et al. 2007; Qi et al. 2013): phenacetin *O*-deethylation for CYP1A2, testosterone 6β-hydroxylation for CYP3A4, coumarin 7-hydroxylation for CYP2A6, chlorzoxazone 6-hydroxylation for CYP2E1, dextromethorphan *O*-demethylation for CYP2D6, diclofenac 4′-hydroxylation for CYP2C9, *S*-mephenytoin 4-hydroxylation for CYP2C19, and paclitaxel 6α-hydroxylation for CYP2C8. All incubations were performed in triplicate, and the mean values were utilized. The typical incubation systems contained 100 mM potassium phosphate buffer (pH 7.4), NADPH generating system (1 mM NADP^+^, 10 mM glucose-6-phosphate, 1 U/mL of glucose-6-phosphate dehydrogenase, and 4 mM MgCl_2_), the appropriate concentration of HLM, a corresponding probe substrate and KF (or positive inhibitor for different probe reactions) in a final volume of 200 μL.

**Table 1.** Isoforms tested, marker reactions, incubation conditions, and *K_m_* used in the inhibition study.

CYPs	Marker reactions	Substrate concentration (μM)	Protein concentration (mg/mL)	Incubation time (min)	Estimated K_m_ (μM)
1A2	phenacetin *O*-deethylation	40	0.2	30	48
3A4	testosterone 6β-hydroxylation	50	0.5	10	53
2A6	coumarin 7-hydroxylation	1.0	0.1	10	1.5
2E1	chlorzoxazone 6-hydroxylation	120	0.4	30	126
2D6	dextromethorphan *O*-demethylation	25	0.25	20	4.8
2C9	diclofenac 4′-hydroxylation	10	0.3	10	13
2C19	*S*-Mephenytoin 4-hydroxylation	100	0.2	40	105
2C8	paclitaxel 6α-hydroxylation	10	0.5	30	16

The concentration of KF was 100 μM according literatures (Lang et al. 2016; Liu et al. 2017; Zhang et al. 2019), and the positive inhibitor concentrations were as follows: 10 μM furafylline for CYP1A2, 1 μM ketoconazole for CYP3A4, 10 μM tranylcypromine for CYP2A6, 50 μM clomethiazole for CYP2E1, 10 μM quinidine for CYP2D6, 10 μM sulfaphenazole for CYP2C9, 50 μM tranylcypromine for CYP2C19, 5 μM montelukast for CYP2C8. Probe substrates, positive inhibitors (except for dextromethorphan and quinidine which were dissolved in water) and KF were dissolved in methanol, with a final concentration of 1% (v/v), and 1% neat methanol was added to the incubations without inhibitor. The final microsomal protein concentration and incubation times for the different probe reactions are shown in Table 1. There was a 3-min preincubation period (at 37 °C) before the reaction was initiated by adding a NADPH-generating system. The reaction was terminated by adding a 100 μL acetonitrile (10% trichloroacetic acid for CYP2A6) internal standard mix, and the solution was placed on ice. The mixture was centrifuged at 12,000 rpm for 10 min, and an aliquot (50 μL) of supernatant was transferred for HPLC analysis. The instrument used in this study were Agilent 1260 series instrument with DAD and FLD detector, and the quantitative assay for the corresponding metabolites was performed as previously reported (Lang et al. 2016; Zhang et al. 2016).

### Enzyme inhibition and kinetic studies of KF

A 100 μM KF was used to initially screen for its direct inhibitory effects toward different human CYP isoforms. For the CYP isoforms whose activities were strongly inhibited, secondary studies were performed to obtain the half inhibition concentration (IC_50_). *Ki* values were obtained by incubating various concentrations of different probe substrates (20–100 μM phenacetin, 20–100 μM testosterone, 2–20 μM diclofenac) in the presence of 0–50 μM KF.

### Time-dependent inhibition study of KF

To determine whether KF could inhibit the activity of CYP1A2, 3A4, and 2C9 in a time-dependent manner, KF (20 μM) was pre-incubated with HLMs (1 mg/mL) in the presence of an NADPH-generating system for 30 min at 37 °C. After incubation, an aliquot (20 μL) was transferred to another incubation tube (final volume 200 μL) containing an NADPH-generating system and probe substrates whose final concentrations were approximate to *K_m_* (Zhang et al. 2017; Dong et al. 2018). Then, further incubations were performed to measure the residual activity. After being incubated for 10 and 30 min, the reactions were terminated by adding a 100 μL acetonitrile internal standard mix and then placed on ice; the corresponding metabolites was determined by HPLC.

To determine the *K_I_* and *k_inact_* values for the inactivation of CYP3A4, the incubations were conducted using higher probe substrate concentrations (approximately 4-fold *K_m_* values) and various concentrations of KF (0–50 μM) after different preincubation times (0–30 min), with a two-step incubation scheme, as described above.

### Statistical analysis

The enzyme kinetic parameters for the probe reaction were estimated from the best fit line using least-squares linear regression of the inverse substrate concentration versus the inverse velocity (Lineweaver-Burk plots), and the mean values were used to calculate Vmax and Km. The equation for competitive inhibition, noncompetitive, time-dependent inhibitions, and inactivation kinetic parameters were used as reported previously (Zhang et al. 2007; Qi et al. 2013). The mechanism of the inhibition was inspected using the Lineweaver-Burk plots and the enzyme inhibition models. The data comparison was performed using Student’s t-test and performed using IBM SPSS statistics 20 (SPSS Inc.).

## Results

**Figure 2.**
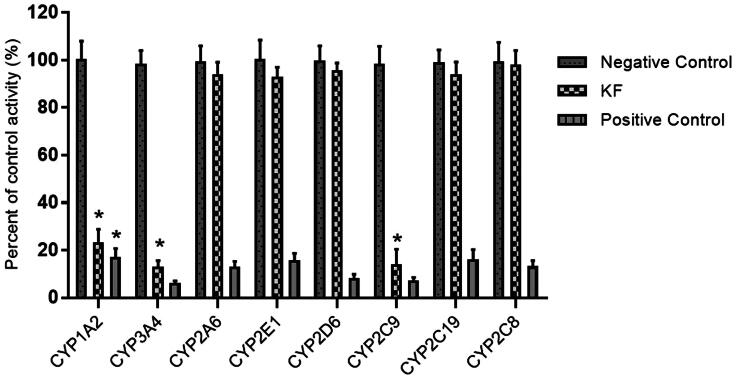
Inhibition of KF on CYP450 enzymes in pooled HLMs. All data represent mean ± S.D. of the triplicate incubations. *p < .05, significantly different from the negative control. Negative control: incubation systems without KF; KF: incubation systems with KF; Positive control: incubation systems with their corresponding positive inhibitors.

To investigate whether the KF affects the catalytic activity of CYP enzymes, the probe reaction assays were conducted with varied concentrations of KF. Specific inhibitors of CYP1A2, 3A4, 2A6, 2E1, 2D6, 2C9, 2C19 and 2C8 were used as positive controls. As shown in Figure 2, KF did not inhibit the activities of CYP2A6, 2E1, 2D6, 2C19 and 2C8 at a concentration of 100 μM. In contrast, the activities of CYP1A2, 3A4, and 2C9 were inhibited to 21.8, 12.7, and 12.9% of their control activities, respectively.

The enzyme-inhibition study showed that inhibition of CYP1A2, 3A4, and 2C9 by KF was concentration-dependent, with IC_50_ values of 20.56, 13.87, and 14.62 μM, respectively.

**Figure 3.**
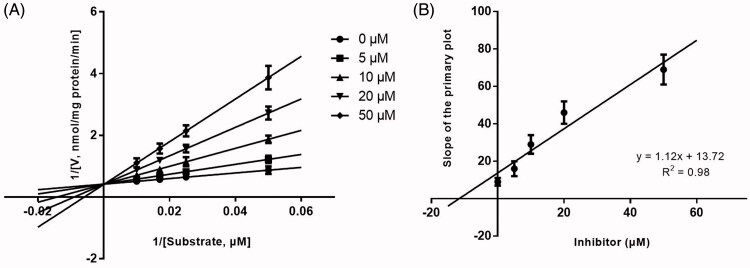
Lineweaver-Burk plots (A) and the secondary plot for *Ki* (B) of inhibition of KF (0–50 μM) on CYP1A2 catalyzed reactions (phenacetin O-deethylation) in pooled HLM. Data are obtained from a 30 min incubation with phenacetin (20–100 μM) in the absence or presence of KF (0–50 μM). The data represent the mean of the incubations (performed in triplicate).

**Figure 4.**
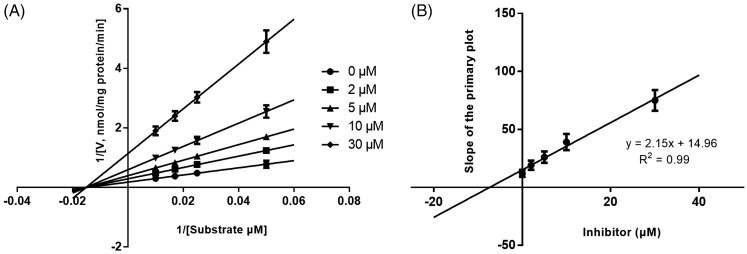
Lineweaver-Burk plots (A) and the secondary plot for *Ki* (B) of inhibition of KF on CYP3A4 catalyzed reactions (testosterone 6β-hydroxylation) in pooled HLM. Data are obtained from a 30 min incubation with testosterone (20–100 μM) in the absence or presence of KF (0–30 μM). All data represent the mean of the incubations (performed in triplicate).

**Figure 5.**
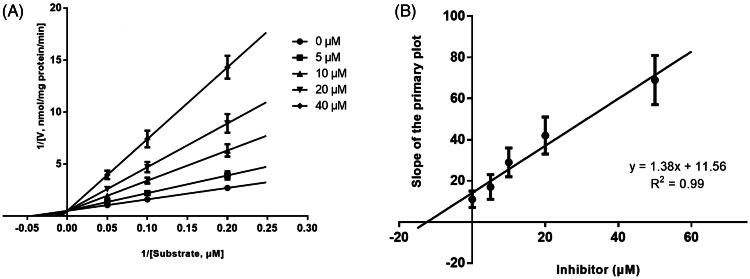
Lineweaver-Burk plots (A) and the secondary plot for *Ki* (B) of inhibition of KF on CYP2C9 catalyzed reactions (diclofenac 4'-hydroxylation) in pooled HLM. Data are obtained from a 30 min incubation with diclofenac (2–20 μM) in the absence or presence of KF (0–40 μM). All data represent the mean of the incubations (performed in triplicate).

Lineweaver-Burk plots of inhibitory kinetic data suggested that the inhibition of CYP1A2 (Figure 3A) and 2C9 (Figure 5A) by KF was best fit in a competitive manner, whereas the inhibition of CYP3A4 by KF was best fit in a noncompetitive manner (Figure 4A). The *Ki* values of KF on CYP1A2 (Figure 3B), 3A4 (Figure 4B), and 2C9 (Figure 5B) were obtained from the secondary Lineweaver-Burk plot for *Ki*, with values of 10.24, 7.11, and 7.58 μM, respectively.

**Figure 6.**
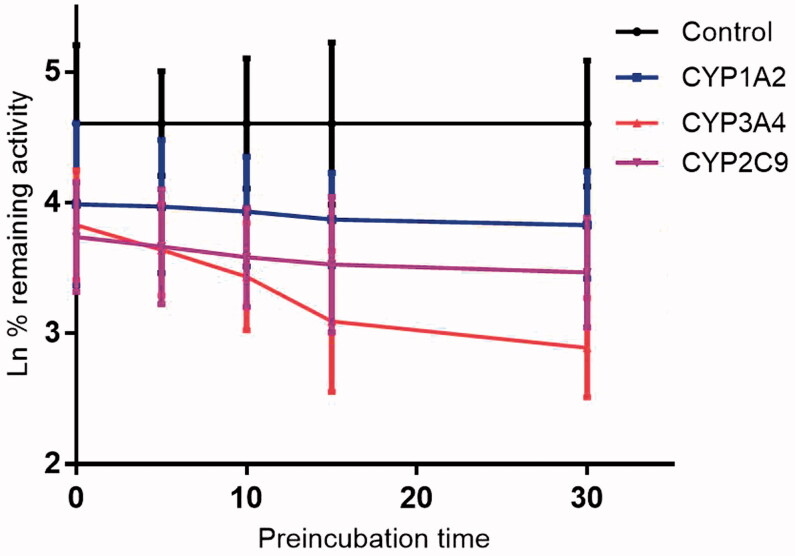
Time-dependent inhibition investigations of CYP1A2, 3A4, and 2C9 catalyzed reactions by KF (20 μM). All data represent the mean of the incubations (performed in triplicate).

**Figure 7.**
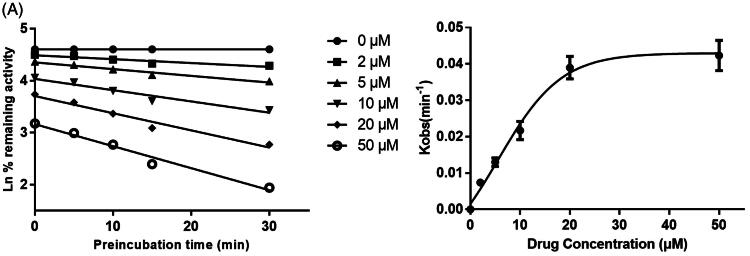
Time and concentration-inactivation of microsomal CYP3A4 activity by KF in the presence of NADPH. The initial rate constant of inactivation of CYP3A4 by each concentration (*K_obs_*) was determined through linear regression analysis of the natural logarithm of the percentage of remaining activity versus pre-incubation time (A). The *K_I_* and *K_inact_* values were determined through non-linear analysis of the *K_obs_* versus the KF concentration (B).

As shown in Figure 6, after pre-incubation of KF with HLM for 30 min, the activity of CYP3A4 decreased with the incubation time, however, the activity of CYP1A2 and 2C9 was not affected. To characterize the time-dependent inhibition of CYP3A4 by KF, inactivation parameters of *K_I_* and *K_inact_* values were calculated using non-linear regression analysis in HLM. As calculated from the inactivation plot of Figure 7, the *K_I_*/*K_inact_* value for CYP3A4 was 10.85/0.036 min/μM. The *K_inact_* values imply that approximately 3.6% of CYP3A4 is inactivated each minute when a saturating concentration of KF is incubated with HLM.

## Discussion

In clinical practice, many patients undergo multiple-drug therapy. Multiple-drug therapy possess several advantages, such as simultaneously treatment of diseases, or multi-drug therapy for the treatment complex chronical disorders, resulting in a better treatment outcome compared to monotherapy. However, many herb-drug interactions resulting from concurrent use of herbal drugs with prescription and over-the-counter drugs may cause adverse reactions such as toxicity and treatment failure (Zhou et al. 2004). The most common causes of herb-drug interactions are modification of the enzyme activity of cytochrome P450 enzymes, specifically through inhibitory effects. Inhibition of CYP enzymes *in vivo* may result in unexpected elevations in the plasma concentrations of concomitant drugs, leading to advievrose effects (Hu et al. 2015; Liu et al. 2015). Therefore, regulatory authorities require preclinical (*in vitro*) and clinical (*in vivo*) interaction studies in the drug development.

As KF possess numerous pharmacological activities, it is essential to investigate the inhibitory effects of KF on the major CYP enzymes. To the best of our knowledge, this study is the first to investigate the effects of KF on the metabolism of probe substrates of several CYP isoforms, including CYP1A2, 3A4, 2A6, 2E1, 2D6, 2C9, 2C19 and 2C8.

CYP1A2 is one of the major CYP enzymes in the human liver, which accounting for approximately 13% of the total content of this enzyme group (Shimada et al. 1997). CYP1A2 plays an important role in the metabolism of several clinically used drugs and foodborne procarcinogens (Singh et al. 2010; Wang and Zhao 2019). Our study showed that KF competitively inhibited human liver microsomal CYP1A2 activity, with *Ki* and IC_50_ values of 10.24 μM and 20.56 μM, respectively. These results indicated that KF could inhibit the activity of CYP1A2, and therefore, KF should be used carefully with drugs metabolized by CYP1A2 such as clozapine, theophyllin, and amitriptyline in order to avoid possible drug interactions (Jeong et al. 2013). As KF was a weak CYP1A2 inhibitor, and the potential of drug-drug interaction with CYP1A2 would be low. However, drugs, especially those with a narrow therapeutic window, are sensitive. Weak to moderate inhibitors still can have impact on the pharmacokinetics and pharmacodynamics of these drugs, such as warfarin, especially (*R*)-warfarin which is mainly metabolized by CYP1A2 and CYP3A4 (Fang et al. 2010). The potential interactions between KF and common drugs need further evaluation in human clinical trials.

The CYP3A subfamily is one of the dominant CYP enzymes in the liver and extra-hepatic tissues, such as the intestines, and it plays an important role in the oxidation of xenobiotics and contributes to the biotransformation of approximately 60% of currently used therapeutic drugs (Pandit et al. 2011). Human CYP3A4 is one of the most abundant drug-metabolizing CYP isoforms in human liver microsomes, accounting for approximately 40% of the total CYP enzymes (Zhou 2008). In fact, characterization of the CYP3A4 isoform responsible for the metabolism of drugs and herbal constituents is important for identifying potential drug-drug or herb-drug interactions in humans. The present study showed that KF had inhibitory effects *in vitro* on CYP3A4 isoform, with Ki and IC_50_ values of 7.11 μM and 13.87 μM, respectively. The results suggested that KF was also a weak CYP3A4 inhibitor, and the potential of herb-drug interaction with CYP3A4 would also be low. However, the results also indicated that KF is a time-dependent inhibitor for CYP3A4 with *K_I/_K_inact_* value of 10.85/0.036 min/μM, which revealed that KF would inhibit the activity of CYP3A4 with increase of preincubation time of concentration. Therefore, in order to avoid adverse drug interactions, KF should not be used with other drugs metabolized by CYP3A4.

CYP2C9 also plays an important role in the metabolism of many drugs (Lim et al. 2014). Our study showed that KF competitively inhibited human liver microsomal CYP2C9 activity. Therefore, KF should also be used carefully with drugs metabolized by CYP2C9 in order to avoid possible drug interactions even though KF was a inhibitor for these two CYP isoforms.

Considering the inhibitory effects of KF on CYP enzymes, clinical interaction with coadministered drugs that are metabolized by CYP enzymes cannot be excluded. As is reported (Song et al. 2019), inhibition of CYP enzymes is a double-edged sword for patients treated with CYP-substrate drugs. Strong inhibition of CYP enzymes may slow down the metabolism of CYP substrates *in vivo*, which may influence the pharmacokinetic properties of co-administrated drugs and thus bring beneficial effects (such as enhancement of efficacy) or unbeneficial effects (such as herb-drug interactions). As we know, *in vitro* data are essential for understanding a potential enzyme inhibition and DDI *in vivo*. However, an observed *in vitro* inhibition of a CYP enzyme does not mean that the drug will cause clinically relevant interactions. Many other factors might influence drug interactions mediated by CYP inhibition, including the contribution of the hepatic clearance to the total clearance of the affected drug, the fraction of the hepatic clearance which is subject to metabolic inhibition, and the ratio of the inhibition constant (*Ki*) over the *in vivo* concentration of the inhibitor (Ito et al. 1998; Ericsson et al. 2014). Therefore, further *in vivo* system studies are needed to identify the interactions of KF with CYP isoform in humans. To predict the potential risks of herb-drug interactions, the *in vitro* to *in vivo* extrapolation (IVIVE) analyses are required (Kirby and Unadkat 2010).

In conclusion, the effects of KF on the activity of CYP enzymes were systematically investigated. The results showed that KF could inhibit the activity of CYP1A2, 3A4, and 2C9, while the activity of other CYP enzymes were not affected. Therefore, in order to avoid adverse drug interactions, it is recommended that KF should not be used with other drugs metabolized by CYP1A2, 3A4, and 2C9.
